# Single Amino Acid Substitution in the Receptor Binding Domain of Spike Protein Is Sufficient To Convert the Neutralization Profile between Ethiopian and Middle Eastern Isolates of Middle East Respiratory Coronavirus

**DOI:** 10.1128/spectrum.04590-22

**Published:** 2023-02-06

**Authors:** Satoko Sugimoto, Masatoshi Kakizaki, Miyuki Kawase, Kengo Kawachi, Makoto Ujike, Wataru Kamitani, Hiroshi Sentsui, Kazuya Shirato

**Affiliations:** a Department of Virology III, National Institute of Infectious Diseases, Musashimurayama, Tokyo, Japan; b Management Department of Biosafety, Laboratory Animals, and Pathogen Bank, National Institute of Infectious Diseases, Musashimurayama, Tokyo, Japan; c Laboratory of Clinical Research on Infectious Diseases, Department of Pathogen Molecular Biology, Research Institute for Microbial Diseases, Osaka University, Suita, Osaka, Japan; d Faculty of Veterinary Medicine, Nippon Veterinary and Life Science University, Musashino, Tokyo, Japan; e Department of Infectious Diseases and Host Defense, Graduate School of Medicine, Gunma University, Maebashi, Gunma, Japan; f Laboratory of Veterinary Epizootiology, Department of Veterinary Medicine, Nihon University, Fujisawa, Kanagawa, Japan; Wuhan Institute of Virology

**Keywords:** Middle East respiratory syndrome (MERS), MERS coronavirus (MERS-CoV), Ethiopia, dromedary, neutralization, amino acid substitution

## Abstract

Middle East respiratory syndrome coronavirus (MERS-CoV) is a zoonotic virus that causes MERS, which is endemic in the Middle East. The absence of human cases in Africa despite the presence of MERS-CoV suggests virological differences between MERS-CoVs in Africa and the Middle East. In fact, in the laboratory, recombinant MERS-CoV carrying the spike (S) protein of Ethiopian isolates exhibits attenuated properties, being more easily neutralized and replicating slower than viruses carrying the S protein of Middle Eastern isolate, EMC. In this study, to identify the amino acids that define the different virological features between Ethiopian and Middle Eastern MERS-CoVs, neutralization titers and viral replication were evaluated using recombinant MERS-CoVs carrying amino acid substitution(s) in the S protein. A single amino acid difference introduced into the receptor binding domain was sufficient to reverse the difference in the neutralizing properties of the S protein between Ethiopian and Middle Eastern MERS-CoVs. Furthermore, amino acid mutations in the S1 and S2 regions of S protein were collectively involved in slow viral replication. Since even a single amino acid difference in S protein can reverse the viral properties of MERS-CoV, it should be noted that multiple mutations may induce a significant change. Careful monitoring of genetic alterations in MERS-CoVs in Africa is therefore required to detect the emergence of virulent strains generated by a few genetic differences.

**IMPORTANCE** There have been no reported cases of human Middle East respiratory syndrome (MERS) in Africa, despite the presence of MERS coronavirus (MERS-CoV). Previous studies have shown that recombinant MERS-CoV carrying the S protein of an Ethiopian isolate replicated slower and was more easily neutralized relative to MERS-CoV carrying the S protein of a Middle Eastern isolate. In this study, we investigated the amino acid(s) in S protein associated with the different viral characteristics between Ethiopian and Middle Eastern MERS-CoVs. The results revealed that a single amino acid difference in the receptor binding domain was sufficient to reverse the neutralization profile. This implies that slight genetic changes can alter the predominant population of MERS-CoV, similar to the transition of variants of severe acute respiratory syndrome coronavirus-2. Careful genetic monitoring of isolates is important to detect the spread of possible virulent MERS-CoVs generated by mutation(s).

## OBSERVATION

Middle East respiratory syndrome (MERS) is an emerging respiratory disease caused by the MERS coronavirus (MERS-CoV), which has been endemic to Saudi Arabia since 2012 (1). It has remained endemic throughout the severe acute respiratory coronavirus 2 (SARS-CoV-2) pandemic, and, as of 6 October 2022, there have been 2,591 confirmed cases in 27 countries, resulting in 894 deaths (2). The dromedary camel is the primary reservoir of MERS-CoV, and the virus is transmitted to humans through close contact with dromedaries (3, 4). Although the dromedary camels in Northern and Eastern African countries have shown extremely high seropositive rates (5, 6), no human cases have been reported, suggesting differences in the characteristics of the viruses circulating in these regions. We previously reported that a recombinant MERS-CoV carrying the spike (S) protein of Ethiopian isolate showed delayed viral replication and different cross-reactivity on neutralization compared with the virus carrying the S protein of Middle Eastern isolate (EMC, JX869059) (7). This indicated that the determinant(s) of these differences in viral replication and neutralization was located in the S protein; the present study aimed to identify this determinant(s).

In the previous study (7), nasal swabs of dromedaries were obtained in Amibara area in Ethiopia and infection of MERS-CoV were confirmed by direct fluorescent RT-LAMP assay (8). The full-length sequence of Amibara isolates were obtained by RNA sequencing with HiSeq X 10 systems (Illumina Inc., San Diego, CA, USA) from MERS-CoV positive specimens, and two sequences were registered to GenBank (MK564474, camel/MERS/Amibara/118/2017; MK564475, camel/MERS/Amibara/126/2017). Recombinant MERS-CoVs were generated using a bacterial artificial chromosome (BAC) clone carrying the full-length infectious genome of the EMC isolate (pBAC-MER-wt) as reported previously (7, 9). Briefly, the S protein sequence of Amibara isolates were synthesized by Strings DNA Fragments service (Thermo Fisher Scientific, Waltham, MA, USA). The S protein sequences of the EMC and Ethiopian (MK564474, camel/MERS/Amibara/118/2017) isolates were cloned into the pKS336 vector. Gene manipulation, such as the insertion of substitutions, was performed on these vectors using site-directed mutagenesis techniques. Then, the S protein sequence on pBAC-MERS-wt was replaced with the mutated EMC or Amibara/118/2017 S protein sequence using a Red/ET recombination system and a counterselection BAC modification kit (Gene Bridges, Heidelberg, Germany). The recombinants were recovered by transfection of BHK cells with BAC plasmid and cocultivation with Vero/TMPRSS2 cells (7).

**FIG 1:**
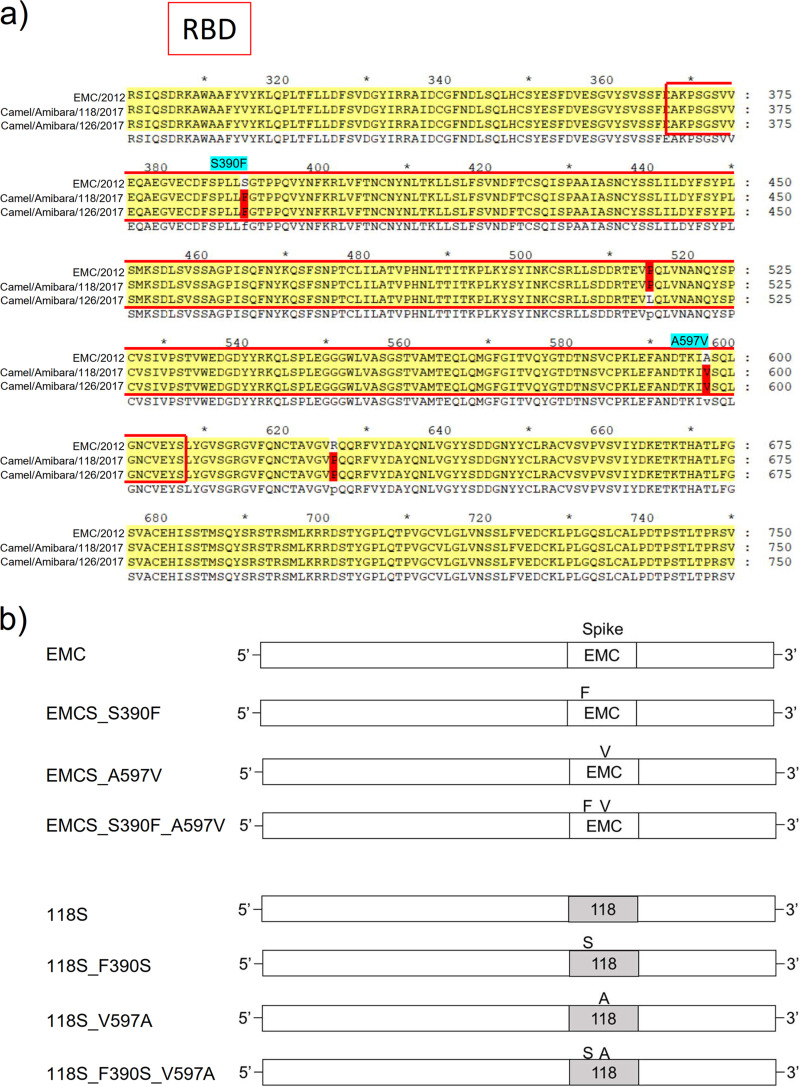

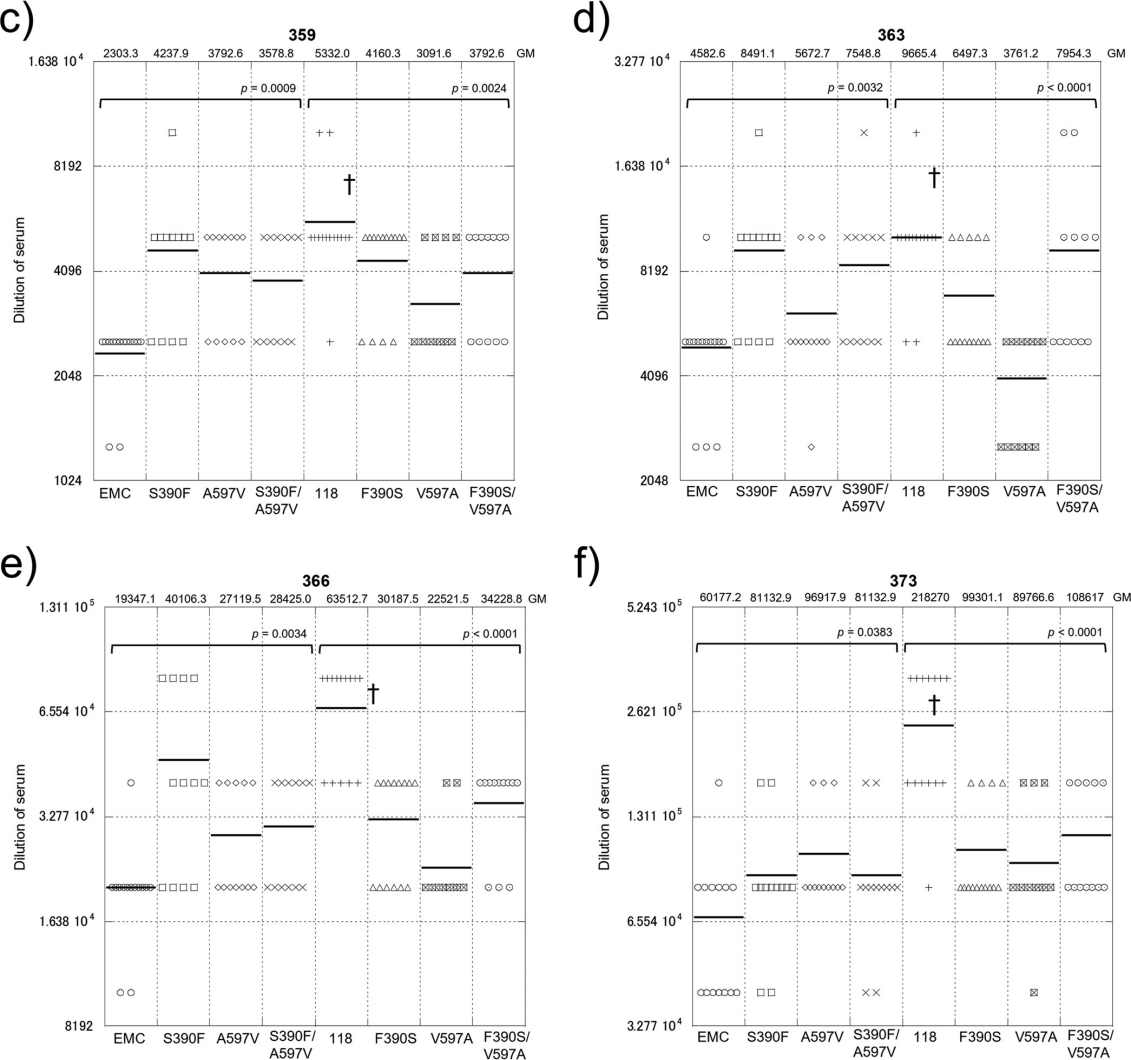
(a) Alignment of the amino acid sequences of the RBD region in the S protein of EMC and Ethiopian MERS-CoVs. The amino acid sequence of S protein (position 300 to 675 for EMC isolate [JX869059]) and the equivalent sequences of Ethiopian MERS-CoVs (Amibara/118 [MK564474] and Amibara/126 [MK564475]) were aligned using MEGA11 software (23). The RBD is indicated by a red line. Amino acid differences between EMC and Ethiopian MERS-CoVs are colored in red. Common differences are highlighted in light blue. (b) Schematic images of recombinant viruses. The recombinant viruses were constructed based on the sequences of EMC isolates. Only S proteins were replaced as previously described (7). (c–f) Neutralizing assay using recombinant viruses. The serum from four different camels (numbers 359, 363, 366, and 373) were used. Neutralizing assay was performed on Vero/TMPRSS2 cells as previously described (7) (*n* = 12). †: Recombinant virus carrying Amibara/118 S protein showed higher neutralizing titer than that of EMC S protein (*P < *0.005). The *P* values calculated by Kruskal-Wallis analysis were shown in each graph and geometric means were shown above each column.

Our previous study indicated that the determinant(s) of the differences in cross-reactivity for neutralization were located in the S1 region of S protein, which is involved in viral host receptor recognition, by experiments involving replacement of the S1 and S2 regions between EMC and Ethiopian isolates (7). There are two common amino acids differences on the receptor binding domain (RBD) of S protein between the EMC and Ethiopian isolates, i.e., S to F at position 390 (S390F) and A to V at position 597 (A597V) of the EMC S protein (Fig. 1a). Therefore, these two substitutions were inserted into the recombinant viruses (Fig. 1b) and their effects on cross-reactivity were assessed by a neutralizing assay using Vero/TMPRSS2 cells (Fig. 1c to f). Four sera (numbers 359, 363, 366, and 373) each obtained from a different dromedary in 2013 (7, 10, 11) were used for the neutralization assay as previously described (7). Unpaired *t* test and Kruskal-Wallis test were used as statistical analyses. Consistent with our previous report (7), the S protein of Amibara/118/2017 reproducibly showed a higher antibody titer than the sera from the EMC isolate of Ethiopian dromedaries (*P < *0.005, Fig. 1c to f). As a side note, the S protein of Ethiopian MERS-CoV also showed a high antibody titer against mouse sera immunized with EMS S protein, suggesting that it is not the locality of virus distribution but the ease of neutralization of S protein by Ethiopian isolates compared with EMS isolates (7). Introduction of single and double amino acid substitutions of S390F and A597V into the EMC S protein generally increased the antibody titer, and thereby the ease of neutralization; whereas, introduction of the reverse substitutions (F390S and V597A) into the Amibara/118 S protein generally decreased the antibody titer, hence making it more difficult to be neutralized. These findings suggested that the molecular determinant of the difference in neutralization between the EMC and Ethiopian S proteins is a single amino acid difference in the RBD region.

**FIG 2:**
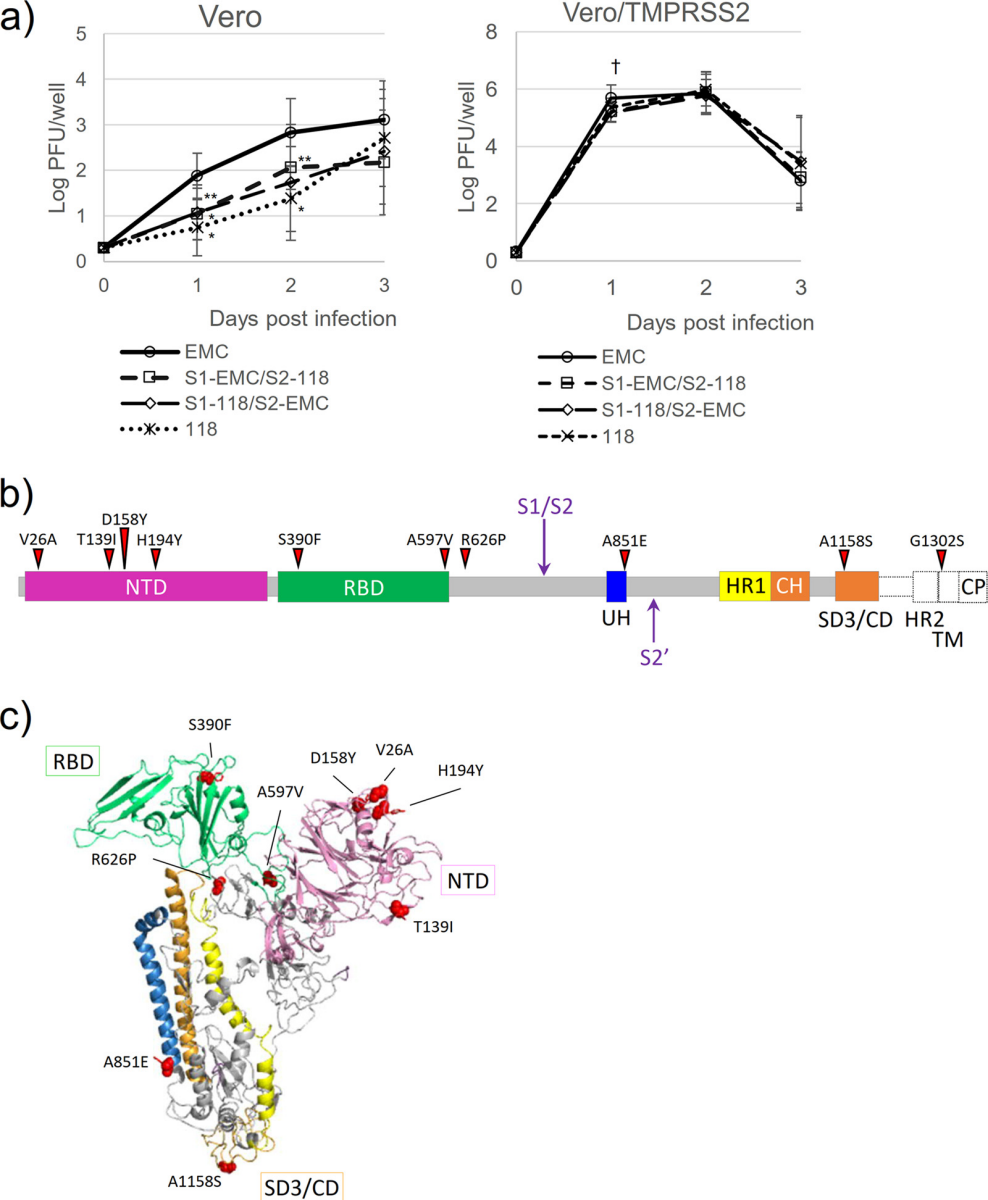
(a) Viral replication of S1/S2 chimeric recombinants. The S1 and S2 regions were replaced between EMC and Amibara/118 on the BAC vector and recombinants were recovered. Viruses [100 PFU] were inoculated onto Vero (*n* = 8) and Vero/TMPPRSS2 (*n* = 3) cells and the supernatants were collected at the indicated time points (0-, 1-, 2-, and 3-days postinoculation). The titer was quantitated by a plaque assay on Vero/TMRPSS2 cells as previously described (7). †: The recombinant virus carrying the EMC S protein showed higher virus replication on day 1 postinoculation compared with the other viruses (*P < *0.005). *, **, Statistical difference in the recombinants carrying the S1/S2 chimeric or Amibara/118 S protein compared with those carrying the EMC S protein. *, *P < *0.01. **; *P* < 0.05. (b) Schematic representation of the MERS-CoV S protein organization. The domains are described based on previous reports (12, 13, 24). Red arrowheads indicate amino acid residues that were different between the EMC S and Amibara/118 S proteins. NTD, N-terminal domain; RBD, receptor-binding domain; S1/S2, S1/S2 cleavage sites; UH, upstream helix; S2′, S2′ cleavage site; HR, heptad repeat; CH, central helix; SD3, subdomain 3; CD, connector domain; TM, transmembrane region/domain; CP, cytoplasmic tail. Dashed lines denote unresolved sequences and regions beyond the construct. (c) Different amino acid residues (red) between the EMC S and Amibara/118 S proteins in a single promoter model of the trimeric MERS-CoV S protein. The abbreviations and colors of elements are the same as in b. The cartoon representation was created by superimposing the sequence of camel/MERS/Amibara/118/2017 S protein (MK564474) with a previously reported structure (PDB: 5X5C) using the SWISS-MODEL server (25) and the PyMOL Molecular Graphics System, version 2.6.0a0 Open-Source (Schrödinger, LLC).

In a previous report, the recombinant viruses carrying the Ethiopian S protein showed slower virus replication as a result of decreased viral entry compared with virus carrying the EMC S protein, might be caused by the difference of preferred entry route (7). To elucidate the molecular determinant(s) of this slower replication, the replication kinetics of an S1/S2 chimeric recombinant between EMC and Amibara/118 were analyzed on Vero and Vero/TMPRSS2 cells (Fig. 2a). Consistent with our previous report (7), the recombinant virus carrying Amibara/118 S protein revealed slower replication than that of EMC S protein on days 1 to 2 postinfection (*P < *0.01). Both the S1/S2 chimeric recombinant viruses (S1-EMC/S2-Amibara/118 and S1-Amibara/118/S2-EMC) showed slower replication relative to recombinant virus carrying the EMC S protein in Vero cells. In Vero/TMPRSS2 cells, the S1/S2 chimeric recombinant viruses showed slower replication on 1 day postinfection, but all recombinant virus reached plateau at same level. These results were identical to a previous report (7), suggesting that the molecular determinant was not a single amino acid substitution, but multiple determinants in each S1 and S2 region. When considering the domains responsible for the differences in virus entry that might decrease the virus replication rate (7), the N-terminal domain (NTD) in S1 and subdomain 3 (12) or the connector domain (13) (SD3/CD) in S2 were identified. The NTD has been reported to interact with host sialic acid (14, 15). Four amino acid differences have been found in NTD (Fig. 2b and c, pink) that might influence binding to host cells. The SD3/CD, which is located proximal to the viral membrane, also possesses a single amino acid difference (Fig. 2b and c, orange). The SD3/CD contains the epitope of neutralizing antibody G4 (13), suggesting the importance of the function of this domain in infection. Although the laboratory strain of human coronavirus 229E (HCoV229E) prefers late endosomal pathway (cathepsin route) and the clinical strain prefers early endosomal pathway (TMPRSS2 route) for cell entry, it has been shown that a single amino acid substitution located proximal to the viral membrane can convert the susceptibility to proteases by modification of exposure of protease recognition site, and affect their viral entry (16). Thus, these mutations in S1 and S2 might collectively influence the virus replication rate (Fig. 2a).

In this study, we showed that the difference in antigenicity between the S proteins of EMC and Ethiopian MERS-CoV isolates was caused by a single amino acid substitution in the RBD region. Including data from a previous study, this difference was found to be several-fold. In influenza virus, it is well known the antigenic “drift” is caused by amino acid substitutions on surface glycoproteins (17). Viruses showing several-fold differences are considered “like” viruses, but the accumulation of these small differences induces large differences that lead to evasion of vaccine immunity (18). The biological significance of such several-fold antigenic differences between coronavirus isolates has remained unknown. However, the emergence of variants of concern (VOC) of SARS-CoV-2 has revealed the importance of these severalfold antigenic differences that have emerged in vaccinated individuals (19, 20), with the dominant VOC being replaced by a newly emerged VOC repeatedly (21). Our study suggests the attenuated properties of Ethiopian MERS-CoV. This implies that if the Middle Eastern strain of MERS-CoV invades Ethiopia, it would quickly spread and become the dominant variant. However, the commercial transportation of dromedaries from Ethiopia to the Middle East is generally one-way (22), so the invasion of Middle Eastern MERS-CoV into Ethiopia via infected dromedaries is not predicted. Continued surveillance of MERS-CoV is important to prevent the spread of pathogenic strains of MERS-CoV to new areas, even during the SARS-CoV-2 pandemic.

## References

[1] Zaki AM, van Boheemen S, Bestebroer TM, Osterhaus AD, Fouchier RA. 2012. Isolation of a novel coronavirus from a man with pneumonia in Saudi Arabia. N Engl J Med 367:1814–1820. doi:10.1056/NEJMoa1211721. 23075143

[2] Regional office for the Eastern Mediterranean WHO. Middle East respiratory syndrome. https://www.emro.who.int/health-topics/mers-cov/mers-cov.html. Accessed 6 October.

[3] Hemida MG, Perera RA, Wang P, Alhammadi MA, Siu LY, Li M, Poon LL, Saif L, Alnaeem A, Peiris M. 2013. Middle East Respiratory Syndrome (MERS) coronavirus seroprevalence in domestic livestock in Saudi Arabia, 2010 to 2013. Euro Surveill 18:20659. 24342517 10.2807/1560-7917.es2013.18.50.20659

[4] Azhar EI, El-Kafrawy SA, Farraj SA, Hassan AM, Al-Saeed MS, Hashem AM, Madani TA. 2014. Evidence for camel-to-human transmission of MERS coronavirus. N Engl J Med 370:2499–2505. doi:10.1056/NEJMoa1401505. 24896817

[5] Muller MA, Corman VM, Jores J, Meyer B, Younan M, Liljander A, Bosch BJ, Lattwein E, Hilali M, Musa BE, Bornstein S, Drosten C. 2014. MERS coronavirus neutralizing antibodies in camels, Eastern Africa, 1983–1997. Emerg Infect Dis 20:2093–2095. doi:10.3201/eid2012.141026. 25425139 PMC4257824

[6] Miguel E, Chevalier V, Ayelet G, Ben Bencheikh MN, Boussini H, Chu DK, El Berbri I, Fassi-Fihri O, Faye B, Fekadu G, Grosbois V, Ng BC, Perera RA, So TY, Traore A, Roger F, Peiris M. 2017. Risk factors for MERS coronavirus infection in dromedary camels in Burkina Faso, Ethiopia, and Morocco, 2015. Euro Surveill 22:30498. 28382915 10.2807/1560-7917.ES.2017.22.13.30498 PMC5388105

[7] Shirato K, Melaku SK, Kawachi K, Nao N, Iwata-Yoshikawa N, Kawase M, Kamitani W, Matsuyama S, Tessema TS, Sentsui H. 2019. Middle East Respiratory Syndrome coronavirus in dromedaries in Ethiopia is antigenically different from the Middle East Isolate EMC. Front Microbiol 10:1326. doi:10.3389/fmicb.2019.01326. 31275264 PMC6593072

[8] Shirato K, Semba S, El-Kafrawy SA, Hassan AM, Tolah AM, Takayama I, Kageyama T, Notomi T, Kamitani W, Matsuyama S, Azhar EI. 2018. Development of fluorescent reverse transcription loop-mediated isothermal amplification (RT-LAMP) using quenching probes for the detection of the Middle East respiratory syndrome coronavirus. J Virol Methods 258:41–48. doi:10.1016/j.jviromet.2018.05.006. 29763640 PMC7113683

[9] Terada Y, Kawachi K, Matsuura Y, Kamitani W. 2017. MERS coronavirus nsp1 participates in an efficient propagation through a specific interaction with viral RNA. Virology 511:95–105. doi:10.1016/j.virol.2017.08.026. 28843094 PMC7118922

[10] Fukushi S, Fukuma A, Kurosu T, Watanabe S, Shimojima M, Shirato K, Iwata-Yoshikawa N, Nagata N, Ohnishi K, Ato M, Melaku SK, Sentsui H, Saijo M. 2018. Characterization of novel monoclonal antibodies against the MERS-coronavirus spike protein and their application in species-independent antibody detection by competitive ELISA. J Virol Methods 251:22–29. doi:10.1016/j.jviromet.2017.10.008. 28993122 PMC7113858

[11] Li TC, Yoshizaki S, Zhou X, Sentsui H, Shirato K, Matsuyama S, Melaku SK, Bazartseren B, Takeda N, Wakita T. 2017. Serological evidence of hepatitis E virus infection in dromedary camels in Ethiopia. J Virol Methods 246:34–37. doi:10.1016/j.jviromet.2017.04.008. 28438608

[12] Yuan Y, Cao D, Zhang Y, Ma J, Qi J, Wang Q, Lu G, Wu Y, Yan J, Shi Y, Zhang X, Gao GF. 2017. Cryo-EM structures of MERS-CoV and SARS-CoV spike glycoproteins reveal the dynamic receptor binding domains. Nat Commun 8:15092. doi:10.1038/ncomms15092. 28393837 PMC5394239

[13] Pallesen J, Wang N, Corbett KS, Wrapp D, Kirchdoerfer RN, Turner HL, Cottrell CA, Becker MM, Wang L, Shi W, Kong WP, Andres EL, Kettenbach AN, Denison MR, Chappell JD, Graham BS, Ward AB, McLellan JS. 2017. Immunogenicity and structures of a rationally designed prefusion MERS-CoV spike antigen. Proc Natl Acad Sci USA 114:E7348–E7357. 28807998 10.1073/pnas.1707304114 PMC5584442

[14] Awasthi M, Gulati S, Sarkar DP, Tiwari S, Kateriya S, Ranjan P, Verma SK. 2020. The sialoside-binding pocket of SARS-CoV-2 spike glycoprotein structurally resembles MERS-CoV. Viruses 12:909. doi:10.3390/v12090909. 32825063 PMC7551769

[15] Park YJ, Walls AC, Wang Z, Sauer MM, Li W, Tortorici MA, Bosch BJ, DiMaio F, Veesler D. 2019. Structures of MERS-CoV spike glycoprotein in complex with sialoside attachment receptors. Nat Struct Mol Biol 26:1151–1157. doi:10.1038/s41594-019-0334-7. 31792450 PMC7097669

[16] Shirato K, Kanou K, Kawase M, Matsuyama S. 2017. Clinical isolates of human coronavirus 229E bypass the endosome for cell entry. J Virol 91:e01387-16. doi:10.1128/JVI.01387-16. 27733646 PMC5165181

[17] Kim H, Webster RG, Webby RJ. 2018. Influenza virus: dealing with a drifting and shifting pathogen. Viral Immunol 31:174–183. doi:10.1089/vim.2017.0141. 29373086

[18] Centers for Disease Control and Prevention. 2021. Understanding flu viruses. https://www.cdc.gov/flu/about/viruses/index.htm. Accessed 14 October 2022.

[19] Kato H, Miyakawa K, Ohtake N, Go H, Yamaoka Y, Yajima S, Shimada T, Goto A, Nakajima H, Ryo A. 2022. Antibody titers against the Alpha, Beta, Gamma, and Delta variants of SARS-CoV-2 induced by BNT162b2 vaccination measured using automated chemiluminescent enzyme immunoassay. J Infect Chemother 28:273–278. doi:10.1016/j.jiac.2021.11.021. 34857462 PMC8627865

[20] Wu J, Nie J, Zhang L, Song H, An Y, Liang Z, Yang J, Ding R, Liu S, Li Q, Li T, Cui Z, Zhang M, He P, Wang Y, Qu X, Hu Z, Wang Q, Huang W. 2022. The antigenicity of SARS-CoV-2 Delta variants aggregated 10 high-frequency mutations in RBD has not changed sufficiently to replace the current vaccine strain. Signal Transduct Target Ther 7:18. doi:10.1038/s41392-022-00874-7. 35046385 PMC8767530

[21] Organization WH. 2022. Tracking SARS-CoV-2 variants. https://www.who.int/activities/tracking-SARS-CoV-2-variants. Accessed 14 October 2022.

[22] Eshetu E, Abraham Z. 2016. Review on live animal and meat export marketing system in Ethiopia: challenges and opportunities. J Sci Innov Res 5:59–64. doi:10.31254/jsir.2016.5206.

[23] Tamura K, Stecher G, Kumar S. 2021. MEGA11: molecular Evolutionary Genetics Analysis Version 11. Mol Biol Evol 38:3022–3027. doi:10.1093/molbev/msab120. 33892491 PMC8233496

[24] Hatmal MM, Alshaer W, Al-Hatamleh MAI, Hatmal M, Smadi O, Taha MO, Oweida AJ, Boer JC, Mohamud R, Plebanski M. 2020. Comprehensive structural and molecular comparison of spike proteins of SARS-CoV-2, SARS-CoV and MERS-CoV, and their interactions with ACE2. Cells 9:2638. doi:10.3390/cells9122638. 33302501 PMC7763676

[25] Waterhouse A, Bertoni M, Bienert S, Studer G, Tauriello G, Gumienny R, Heer FT, de Beer TAP, Rempfer C, Bordoli L, Lepore R, Schwede T. 2018. SWISS-MODEL: homology modelling of protein structures and complexes. Nucleic Acids Res 46:W296–W303. doi:10.1093/nar/gky427. 29788355 PMC6030848

